# Longitudinal Analysis of Biologic Correlates of COVID-19 Resolution: Case Report

**DOI:** 10.3389/fmed.2022.915367

**Published:** 2022-06-15

**Authors:** Natalie Bruiners, Valentina Guerrini, Rahul Ukey, Ryan J. Dikdan, Jason H. Yang, Pankaj Kumar Mishra, Alberta Onyuka, Deborah Handler, Joshua Vieth, Mary Carayannopoulos, Shuang Guo, Maressa Pollen, Abraham Pinter, Sanjay Tyagi, Daniel Feingold, Claire Philipp, Steven K. Libutti, Maria Laura Gennaro

**Affiliations:** ^1^Rutgers New Jersey Medical School, Public Health Research Institute, Newark, NJ, United States; ^2^Department of Medicine, Rutgers New Jersey Medical School, Newark, NJ, United States; ^3^Department of Microbiology, Biochemistry and Molecular Genetics, Rutgers New Jersey Medical School, Newark, NJ, United States; ^4^Center for Emerging and Re-emerging Pathogens, Rutgers New Jersey Medical School, Newark, NJ, United States; ^5^Global Tuberculosis Institute, Rutgers New Jersey Medical School, Newark, NJ, United States; ^6^Cancer Institute of New Jersey, Rutgers University, New Brunswick, NJ, United States; ^7^Department of Pathology and Laboratory Medicine, Rutgers Robert Wood Johnson Medical School, New Brunswick, NJ, United States; ^8^Division of Hematology, Rutgers Robert Wood Johnson Medical School, New Brunswick, NJ, United States; ^9^Department of Surgery, Rutgers Robert Wood Johnson Medical School, New Brunswick, NJ, United States

**Keywords:** severe COVID-19, RNAemia, convalescent plasma therapy, plasma proteomics, single-cell transcriptomics

## Abstract

While the biomarkers of COVID-19 severity have been thoroughly investigated, the key biological dynamics associated with COVID-19 resolution are still insufficiently understood. We report a case of full resolution of severe COVID-19 due to convalescent plasma transfusion. Following transfusion, the patient showed fever remission, improved respiratory status, and rapidly decreased viral burden in respiratory fluids and SARS-CoV-2 RNAemia. Longitudinal unbiased proteomic analysis of plasma and single-cell transcriptomics of peripheral blood cells conducted prior to and at multiple times after convalescent plasma transfusion identified the key biological processes associated with the transition from severe disease to disease-free state. These included (i) temporally ordered upward and downward changes in plasma proteins reestablishing homeostasis and (ii) post-transfusion disappearance of a subset of monocytes characterized by hyperactivated Interferon responses and decreased TNF-α signaling. Monitoring specific dysfunctional myeloid cell subsets in peripheral blood may provide prognostic keys in COVID-19.

## Introduction

Two years into the COVID-19 pandemic caused by the severe acute respiratory syndrome coronavirus 2 (SARS-CoV-2) worldwide research effort has defined the immunopathology of COVID-19 (1, 2). The immune responses associated with severe and fatal COVID-19 manifestations include defective antiviral mechanisms such as type I interferon (IFN) responses (3, 4), imbalanced T cell responses (5, 6), abnormal expansion of potentially dysfunctional B cells (7), exuberant inflammation driven by altered myeloid responses (8, 9), and production of potentially pathogenic antibodies (autoantibodies and/or proinflammatory antibodies) (10–12). Some studies leveraged biosamples collected serially from the same patients, thus providing a dynamic representation of the biologic parameters associated with disease worsening [for example (6)]. Much less attention has been placed on characterizing the biological events associated with the clinical resolution of severe COVID-19, even though understanding disease resolution at the molecular and cellular level might contribute new targets of therapeutic interventions against severe COVID-19.

Here we report the first, in-depth, longitudinal analysis of viral and host biologic correlates of the clinical resolution of COVID-19 in a patient who received convalescent plasma therapy during the first pandemic wave in the United States. We find that disease resolution was associated with temporally ordered upward and downward changes in plasma proteins reestablishing homeostasis. Moreover, a subset of monocytes characterized by hyperactivated IFN responses and decreased TNF-α signaling uniquely disappeared following transfusion, implying a key role of this monocyte population in COVID-19 severity and prognosis. Our report of COVID-19 resolution following convalescent plasma therapy in a patient unable to generate humoral responses to SARS-CoV-2 infection, together with a previous report of convalescent plasma-associated COVID-19 resolution in a patient with humoral immunodeficiency (15), strongly supports the use of well-characterized convalescent plasma for therapeutic use in COVID-19 patients who exhibit humoral immunocompromise due to underlying immunological defects or immunosuppressive therapies.

## Clinical Course and Transfusion Effects on Viral Load and Clinical Parameters

**Figure 1:**
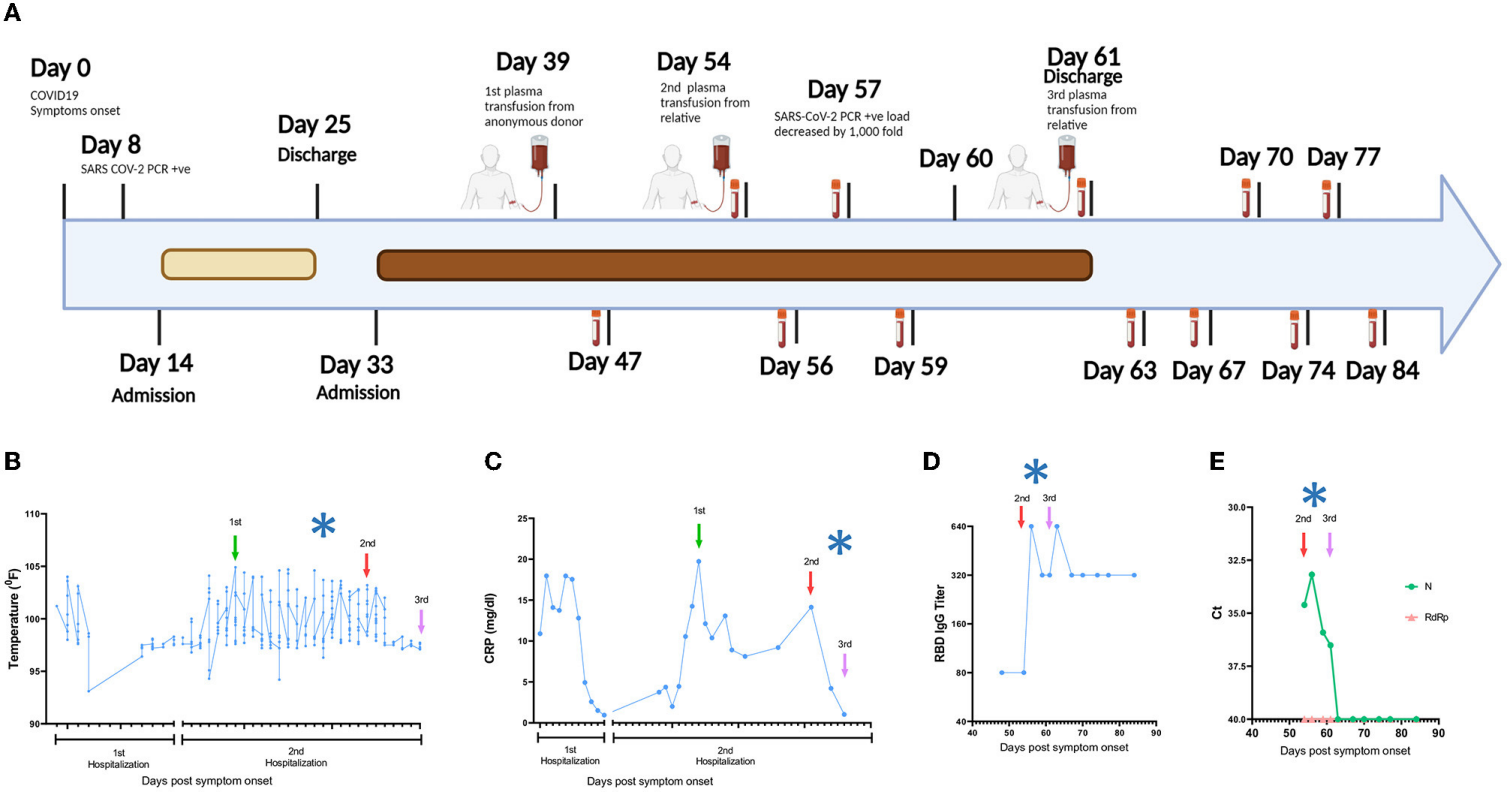
Timeline of the clinical course. **(A)** Day 0 indicates the date of the COVID-19 symptom onset. The days post-onset at which COVID-19 related symptoms, hospitalization course, RT-PCR test results, convalescent plasma transfusions, and interventions are indicated. **(B–E)** Longitudinal analysis of **(B)** maximum body temperature, **(C)** C- Reactive protein (CRP) in plasma, **(D)** Titers of IgG against SARS-CoV-2 Spike Receptor binding domain (RBD), and **(E)** SARS-CoV-2 RNA in plasma. The y axis in each panel indicates the corresponding measurement unit. In all panels, the x axis indicates the day numbering as depicted in **(A)**. In **(B,C)**, the timeline in the x axis highlights the first hospitalization (days 14–25) and the second hospitalization (days 33–61), since the corresponding measurements were performed only in the hospital. In all panels, the vertical arrows indicate convalescent plasma transfusions from anonymous donor (1st) and from a patient's relative (2nd and 3rd). The asterisk indicates the drop in viral load recorded at day 57 **(A)**. The low-level anti-RBD IgG seen pre-transfusion **(D)** are consistent with the lack of B cell detection in the patient's peripheral blood by clinical flow cytometry tests (data not shown), which are presumably due to the use of rituximab and/or methotrexate for MAS, since treatment with these drugs can reduce B cell frequencies and humoral immune responses (13, 14). In **(E)**, Ct, cycle threshold; N, SARS-CoV-2 nucleocapsid gene; RdRP, SARS-CoV-2 RNA-dependent RNA polymerase gene.

In March 2020, a 52-year-old white woman with multiple autoimmune syndrome (MAS), including rheumatoid arthritis and systemic lupus erythematosus developed flu-like symptoms (Figure 1A). Her treatment for MAS included daily hydroxychloroquine, weekly methotrexate, and every 5–6 months Rituximab infusion. Three days after symptom onset, she presented to the emergency department (ED), where the nasopharyngeal fluid PCR test was positive for SARS-CoV-2 and chest X ray (CXR) examination demonstrated early right lower lung infiltrates. She was discharged from the ED with Azithromycin dose pack. On day 14, she returned to the ED with fever (101.2°F) and hypoxia (pulse oximetry of 87% on ambient air). CXR showed bilateral lung opacities with interval progression from the previous exam. She was placed on 4-liter supplemental oxygen and admitted to a hospital with a COVID-19 diagnosis. On day 16, she was started on a 10-day remdesivir course. The hospital course was complicated by hypotension (68/44 mm Hg) and hypothermia on day 17 (93.1°F) (Figure 1B), requiring transfer to the medical intensive care unit and pharmacologic blood pressure support. On day 22, a computed tomography (CT) angiogram of the chest showed bilateral COVID-19 pneumonia and a segmental artery pulmonary embolism; anticoagulation was increased from prophylactic to therapeutic dosing. Fevers resolved, and the patient was weaned off of supplemental oxygen at rest and discharged after completing 10-day course of remdesivir. On day 33, she was re-admitted to the hospital with a fever of 102°F and pulse oximetry of 93% on ambient air. She was diagnosed with severe COVID-19, according to US National Institute of Health classification (https://www.covid19treatmentguidelines.nih.gov/overview/clinical-spectrum/). She was started empirically on supplemental oxygen via nasal cannula, piperacillin/tazobactam, vancomycin, and prednisone. She continued to have high fevers twice daily, accompanied by increasing C-reactive protein (CRP) levels (Figures 1B,C). Given the recent remdesivir course and the recurrence of severe COVID-19 symptoms, the patient received alternative management with 400 mL of COVID-19 convalescent plasma from an anonymous donor on day 40; however, fevers persisted daily reaching maximum temperature of 104.7°F and CT of the chest with contrast on day 45 showed worsened bilateral ground-glass lung opacities compared to 1 week prior. She continued to require supplemental oxygen via nasal cannula (2 liters). On day 48, plasma samples from the patient and family members were analyzed in our research laboratory for antibody testing. Plasma from a patient's relative, who had recovered from COVID-19, showed high anti-SARS-CoV-2 RBD IgG titers (Supplementary Figure S1) and was selected for donation (600 mL; on day 54). Oxygen saturation improved and patient no longer required supplemental oxygen at rest on day 54. Three days later, on day 57, SARS-CoV-2 PCR analysis of a nasopharyngeal sample showed a ~1,000-fold decrease [10 cycle threshold (Ct) values] in viral burden relative to the preceding test result. Concurrently, fevers abruptly resolved on day 57 (Figure 1B) and plasma markers of inflammation, such as CRP, decreased rapidly (Figure 1C). SARS-CoV-2 PCR testing of a nasopharyngeal sample became negative on day 59 and lowly positive on day 60. The patient remained afebrile. Oxygen saturation remained normal at rest. She received a second transfusion of 400 mL of her relative's convalescent plasma on day 61 and was discharged from the hospital without further recurrence of COVID-19 symptoms or re-hospitalization. The SARS-CoV-2 PCR test remained stably negative after this transfusion for >20 days (Figure 1A), after which testing was discontinued. Antibody binding assays performed for >20 days after her discharge showed stable levels of anti-RBD IgG (Figure 1D). The SARS-CoV-2 RNAemia was observed before the beneficial transfusion and became undetectable post-transfusion (Figure 1E). In summary, administration of the relative's convalescent plasma resulted in fever remission, improved respiratory status, return to normal of plasma indicators of inflammation, and drastic decrease of viral RNA levels in plasma and upper respiratory tract fluids.

## Plasma Cytokine and Proteome Self-Organize Temporally and Identify Key Biological Processes During Disease Resolution

**Figure 2:**
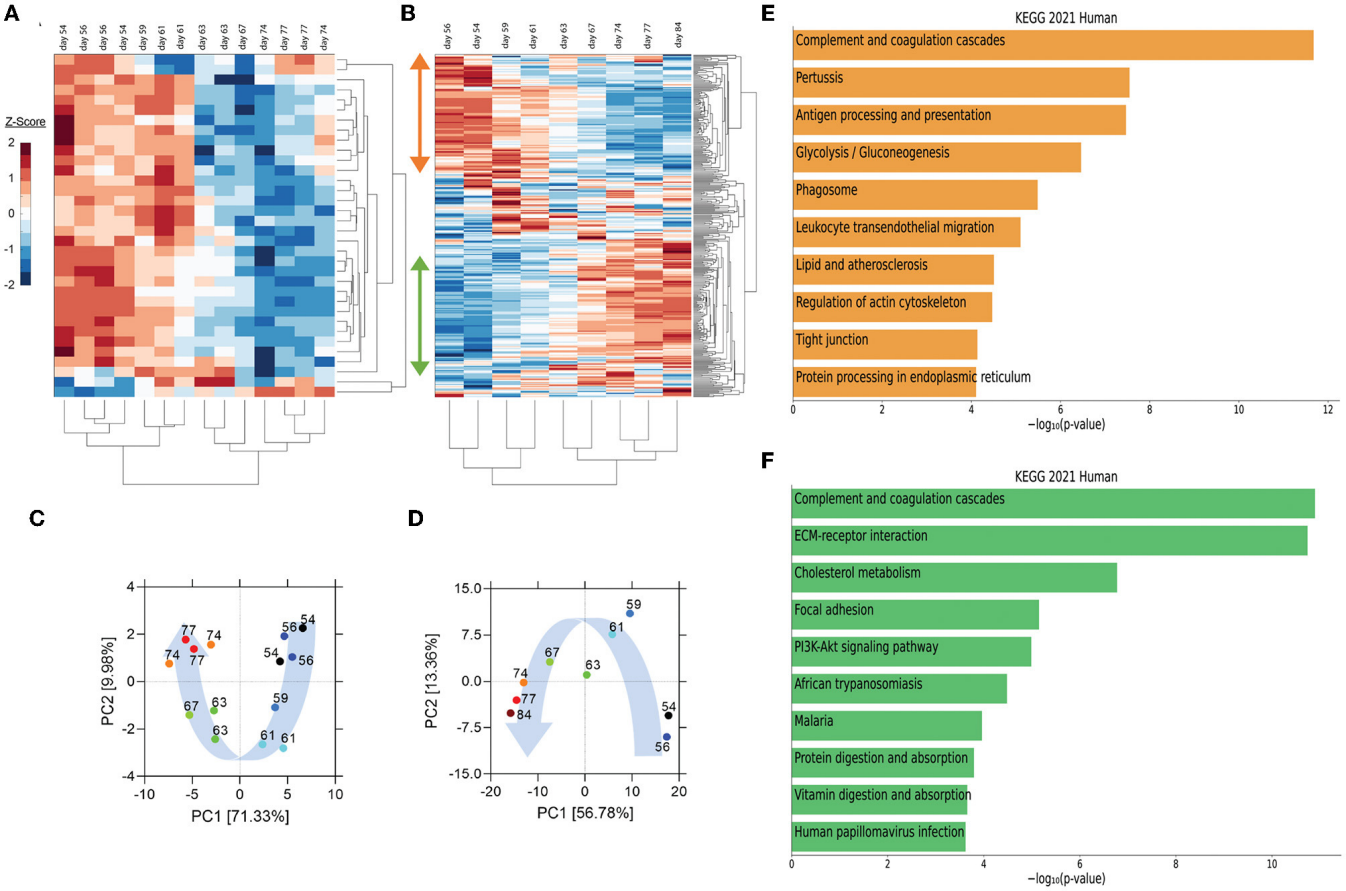
Unsupervised hierarchical clustering and principal component analysis (PCA) of cytokines and proteins in the recipient's plasma before and after convalescent plasma transfusion. **(A,B)** Unsupervised hierarchical clustering was performed on plasma cytokine profile and proteomic data using MATLAB. Vertical arrows in **(B)** mark the set of proteins that decrease (orange arrow) or increase (green arrow) following transfusion. **(C,D)** PCA was conducted on plasma cytokine profile **(C)** and proteome **(D)**. The variance accounted for by PC1 is shown on the x-axis and the variance accounted for by PC2 on the y-axis. Numbers indicate days post-symptom onset as shown in **(A,B)**. Blue arrows illustrate the distribution of PC1 scores, which were self-organized in a temporal trajectory. **(E,F)** The top 10 enriched KEGG pathways for proteins that decrease (orange bars) (referred to as module A in Supplementary Figure S2 and in the text) or increase (green bars) (module B in Supplementary Figure S2 and in the text) using EnrichR Pathway Analysis.

Utilizing peripheral blood samples collected from the patient at multiple times pre- and post-transfusion with her relative's plasma (as indicated in Figure 1A), we performed cytokine and proteomic analysis of plasma collected from the patient collected from the patient on the day of (prior to) the first transfusion (i.e., day 54 post-symptom onset), and multiple times thereafter (see Figure 1A for blood collection points). Unbiased hierarchical clustering analysis and principal component analysis revealed dynamic changes in plasma cytokine and protein concentrations over four temporal phases following transfusion (Figures 2A–D). Changes in the abundance of plasma proteins became detectable between days 56 and 61 (Figures 2A,B)—a few days after the transfusion, and slightly preceded those in plasma cytokines. Between days 61 and 67, the concentration of most plasma cytokines decreased sharply, showing that disease resolution was linked with countering the hyperproduction of inflammatory cytokines typically associated with severe COVID-19 (6). Plasma proteins exhibited a more complex behavior, with some proteins decreasing (Figure 2B, orange vertical arrow) and others increasing (Figure 2B, green vertical arrow) following transfusion. We call these two protein subsets Module A and Module B, respectively (see Supplementary Figure S2 for contribution to Principal Component 1 of the two Modules). By day 74 post-transfusion, plasma cytokines and proteins reached their steady-state concentrations, indicating therapeutic resolution. Bioinformatic analyses of plasma proteins in Modules A and B are presented below.

Pathway enrichment analysis using the Enrichr tool (16) identified “complement and coagulation cascades” as top term in both Module A and Module B (Figures 2E,F), reflecting the known complement and coagulation disorders associated with severe disease by SARS-CoV-2 and other pathogenic coronaviruses (17, 18). Proteins included in this term in Module A (Figure 2E) were predominantly components of the complement cascade (Table 1), consistent with complement hyperactivation potentially causing host tissue damage and disease (19) and being associated with severe COVID-19 (20). The second top term in Module A is “pertussis” (Figure 2E). In addition to proteins also present in “complement and coagulation cascades”, this pathway included cluster of differentiation 14 (CD14) (Table 1). Soluble CD14 (sCD14) and sCD163, which we also find decreased in the patient's plasma following transfusion, contribute to monocyte-macrophage activation and increase with COVID-19 severity (21). Indeed, CD14 is currently being explored as a COVID-19 therapeutic target (clinical trial NCT04391309). The third pathway in Module A was “antigen processing and presentation” (Figure 2E). This pathway includes heat shock proteins (HSP) (Table 1) that have been described as “facilitators” of infections with several viruses, including coronaviruses and Zika (22, 23). Some, such as HSP90, have been proposed as targets of therapeutic strategies against viral replication (24, 25). The “antigen processing and presentation” pathway also included human leukocyte antigen (HLA) class I molecules and β2-microglobulin (β2-m), which is essential for the conformation of the major histocompatibility complex (MHC) class I protein complex (Table 1). Blood levels of soluble HLA antigens are elevated during chronic infection with hepatitis virus B and C and decrease following treatment of chronic viral infections (26), suggesting an association with viral persistence. Moreover, high β2-m levels in blood have been associated with several viral infections, including COVID-19 (27, 28). An additional member of the “antigen processing and presentation” pathway is Calreticulin (Table 1), a molecular chaperone ensuring proper folding and functioning of proteins in the endoplasmic reticulum (ER) (29). Since all coronaviruses use the ER to replicate, severe infection with these viruses and the consequent massive production of viral proteins may induce ER stress and activate a compensatory mechanism known as the unfolded protein response, which can further enhance viral replication (30). Taken together, the analysis of the top three terms in Module A shows that clinical resolution of COVID-19 due to convalescent plasma transfusion was accompanied by the longitudinal decrease of plasma markers associated with pathogenic or harmful functions. These included (i) dampening of the excessive activation of the complement cascade and hyperinflammatory responses, with consequent reduction of their tissue- and organ-damaging effects, and (ii) decreased expression of antigen presentation and various protein modifying systems that may favor viral replication.

**Table 1:** Proteins in the top three KEGG pathways that decrease (module A) or increase (module B) post-transfusion.

		**Kegg term**	**Symbol (name)**
	Post-transfusion decrease	Complement and coagulation cascades	C4A (complement component 4A); C5 (complement component 5); SERPINA1 (alpha-1 antitrypsin); CFHR1 (complement factor H related 1); C9; CFHR3 (complement factor H related 3); CFHR5 (complement factor H related 5); SERPING1 (C1 inhibitor); C4BPA (complement component 4 binding protein alpha); C4BPB (complement component 4 binding protein beta)
Module A		Pertussis	C4A (complement component 4A); C5 (complement component 5); CFL1 (COFILIN 1); SERPING1 (C1 inhibitor); C4BPA (complement component 4 binding protein alpha); C4BPB (complement component 4 binding protein beta); CD14 (cluster of differentiation 14)
		Antigen processing and presentation	HSPA8 (heat shock protein family a member 8); HSP90AB1 (heat shock protein 90 alpha family class B member 1); HSPA1L (heat shock protein family A member 1 Like); HLA-B (human leukocyte antigen B); HLA-A (human leukocyte antigen A); CALR (calreticulin); B2M (beta-2-microglobulin)
	Post-transfusion increase	Complement and coagulation cascades	C7 (complement 7); SERPINC1 (serpin Family C Member 1); F12 (coagulation factor XII); PLG (plasminogen); F13B (coagulation factor XIII B Chaign); A2M (alpha 2-macroglobulin); CLU (clusterin)
Module B		ECM-receptor interaction	COMP (cartilage oligomeric matrix protein); TNXB (tenascin-X); COL1A2 (collagen type I alpha 2 chain); LAMA2 (laminin subunit alpha-2); COL6A1 (collagen, type VI, alpha 1); COL4A5 (collagen Type IV alpha 5 chain); COL6A3 (collagen type VI alpha 3 chain); HSPG2 (heparan sulfate proteoglycan 2); CD44 (cluster of differentiation 44); THBS4 (thrombospondin 4)
		Cholesterol metabolism	LRP1 (lipoprotein receptor-related protein 1); APOH (Apolipoprotein H); APOC1 (Apolipoprotein C-I); APOA1 (apolipoprotein A-I); APOC3 (Apolipoprotein C-III); APOA4 (Apolipoprotein A4)

As mentioned above, the top term in the pathway analysis in Module B, which increased with convalescent plasma transfusion, was also “complement and coagulation cascades” (Figure 2F). Proteins assigned to this term in Module B included mostly coagulation factors that were low prior to the convalescent plasma transfusion, and serine protease inhibitors (Table 1). In addition, this pathway included components of the kallikrein-kinin system (Table 1), which participates in coagulation and control of blood pressure and may contribute to lung angioedema in severe COVID-19 (31). The second pathway in Module B is “Extracellular matrix (ECM) receptor interaction” (Figure 2F; Table 1). Interactions between ECM constituents and cells, which are typically mediated by transmembrane molecules, contribute to the regulation of key cellular functions, including adhesion, migration, and differentiation (32). Proteins associated with ECM receptors and focal adhesion (the fourth topmost pathway in Module B, Figure 2F) have been found decreased in the lung tissue of patients deceased with COVID-19 pneumonia, suggesting dysregulation of the lung extracellular microenvironment (33). The observed increased plasma levels of these proteins following convalescent plasma transfusion—if they reflect changes occurring also in organ microenvironments—could then be considered a meaningful indication of COVID-19 resolution. Furthermore, Module B contains proteins in the “cholesterol metabolism” pathway (Figure 2F; Table 1), which participate in high-density lipoprotein (HDL) cholesterol metabolism (34, 35). These data are consistent with a report of low HDL cholesterol levels in patients with severe COVID-19 (36). Taken together, the longitudinal increase in protein sets in Module B during disease resolution point to key organ and cellular functions required to reestablish tissue homeostasis and a disease-free state.

## Convalescent Plasma Transfusion Is Associated With the Disappearance of Dysfunctional Monocytes

**Figure 3:**
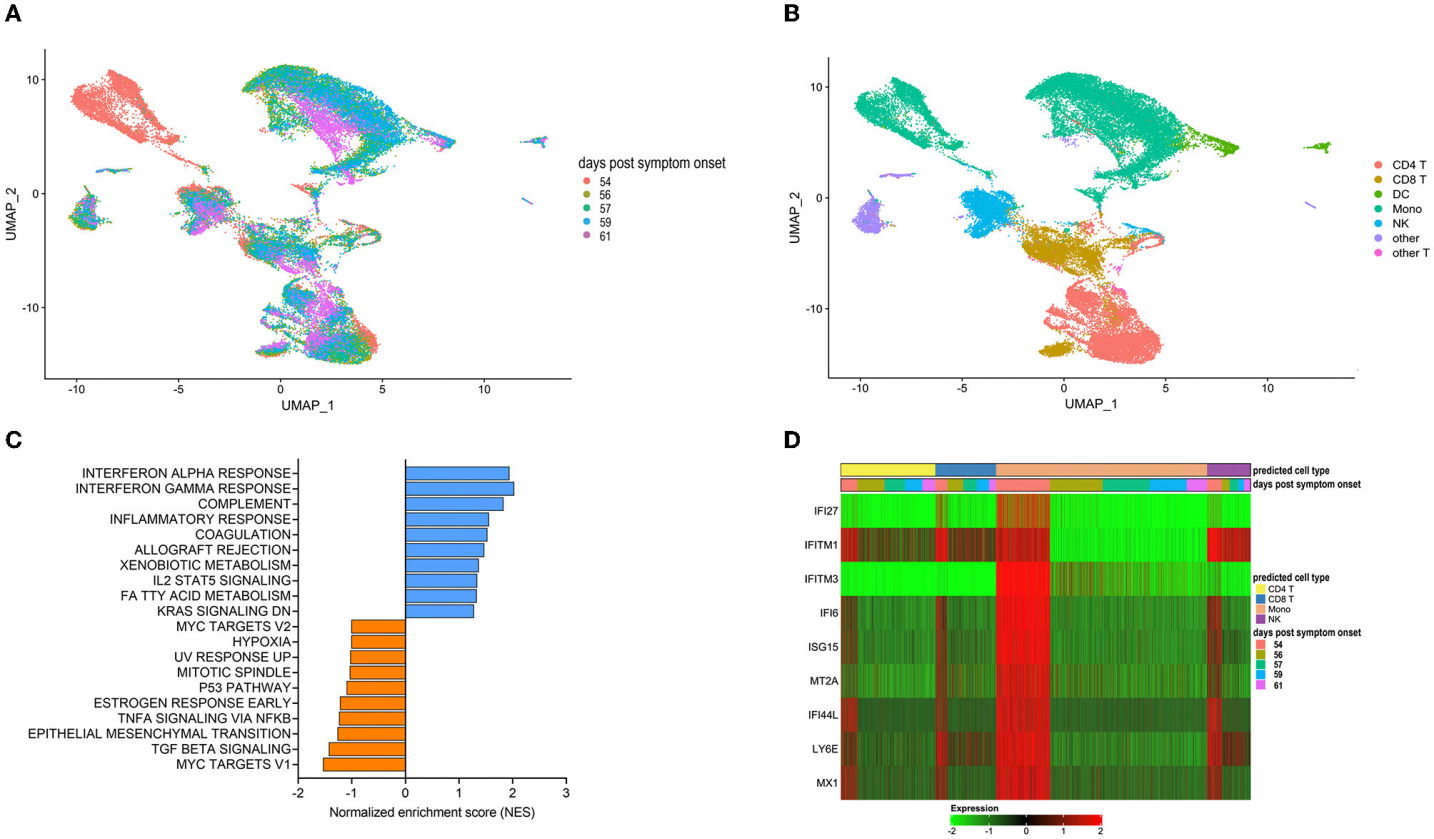
Single cell transcriptomics of recipient's peripheral blood mononuclear cells before and after convalescent plasma transfusion. **(A)** Uniform Manifold Approximation and Projection (UMAP) distribution of single cells grouped by time-post-transfusion sample. Day 0 is the sample obtained just before the first transfusion with the relative's convalescent plasma. **(B)** Cell types were determined using Seurat's MapQuery function along with their PBMC reference dataset. It is worth noting that none of the cell types identified in **(B)** could be assigned to B cells, both prior to the transfusion and for the duration of the post-transfusion analysis, in agreement with flow cytometry data recorded in the patient's hospital chart, as mentioned in the legend to Figure 1. **(C)** Histogram of Hallmark gene set enrichment analysis (GSEA) using differentially expressed genes of the unique monocyte cluster identified at day 0 compared to monocytes across other data clusters. **(D)** Heatmap showing the expression levels of type I Interferon-regulated genes in predicted T cells, monocytes, and NK cells. The color code of the “days post-transfusion” bar corresponds to the color code in **(A)**.

We next utilized single-cell RNA sequencing to analyze transcriptional changes occurring in peripheral blood cells collected from the patient on the day of (prior to) the first transfusion with her relative's plasma (i.e., day 54 post-symptom onset), and multiple times thereafter (see Figure 1A for blood collection points). Unsupervised clustering identified one data cluster that was observed pre-transfusion but was absent at subsequent days (Figure 3A). Cell type identification analysis using the Seurat package (37) identified this data cluster as being constituted by monocytes (Figure 3B). We next performed Hallmark gene set enrichment analysis to identify genes that best distinguished the monocyte cluster unique to the pre-transfusion state from monocytes in other data clusters. The top hits among upregulated genes [positive normalized enrichment score (NES)] included genes involved in responses to interferon (IFN) alpha (type I), IFN gamma (type II), inflammatory responses, and complement and coagulation (Figure 3C). While type I IFNs are key mediators of antiviral responses (38), their excessive activation can exacerbate hyperinflammation and the associated severe manifestations of COVID-19 (39). Similar considerations can be made for type II IFN, which may be important for antiviral defense but may worsen systemic inflammation and increase organ damage when produced at persistently high levels (38). Thus, the hyperactivation of IFN-γ observed in this patient may have contributed to hyperinflammation and COVID-19 severity. The top hits among downregulated genes (negative NES) comprised MYC targets and signaling by cytokines such as TGF-β and TNF-α (Figure 3C). The downregulation of MYC targets is difficult to interpret given the highly diverse cellular functions of the ~ 400 genes in this gene set. The finding that TNF-α signaling is decreased in the monocyte cluster unique to the pre-transfusion state leads to two sets of considerations. First, the pre-transfusion high levels of TNF-α in plasma (data included in Figure 2A) were likely produced by cell types other than this unique monocyte cluster. Second, an imbalance between signaling by type I IFN (increased) and TNF-α (decreased) in monocytic cells, as observed in this patient, has been proposed to contribute to loss of protective innate immune functions in hyperinflammatory or autoimmune conditions (40) and has been described in severe COVID-19 (41). Of note, upregulation of both type I IFN and TNF-α responses (42) and imbalances in the opposite direction (dampened type I IFN and elevated TNF-α signaling) (43, 44) have also been reported in severe COVID-19, suggesting that immune dysregulation phenotypes may vary in different patients, presumably depending on context. Furthermore, when we analyzed individual Interferon stimulated gene (ISG) expression, we observed that typical ISGs, such as IFI6, ISG15, IFI44L, and LY6E were most prominently elevated in monocytes prior to transfusion but were also increased in NK cells and T cell subsets, and that this strong IFN response was dampened post-transfusion also in these cell types (Figure 3D). Together, these data show that the beneficial effects of the relative's plasma transfusion were accompanied by the elimination of a unique population of disease-associated, hyperinflammatory monocytes.

## Discussion

The present report constitutes, to our knowledge, the first longitudinal molecular and cellular in-depth analysis of COVID-19 resolution. In the study patient, severe COVID-19 was associated with SARS-CoV-2 RNAemia, high levels of pro-inflammatory mediators and other potentially harmful plasma factors, dysregulation of coagulation cascades, and abnormally low levels of proteins related to systemic and tissue homeostasis. Following convalescent plasma transfusion from a family member, the plasma protein landscape was characterized by temporally organized clusters of cytokines and other plasma proteins, indicative of dynamic trajectories toward disease resolution. Disease resolution was also associated with post-transfusion disappearance of monocytes characterized by hyperactivation of type I and type II IFN cascades and dampening of TNF-α signaling. Even though we cannot rigorously ascribe disease resolution to the relative's convalescent plasma transfusion, the temporal relationship between transfusion and the dramatic improvement of all virologic and clinical parameters, which had not occurred with any of the previous empirical treatments, is compelling. Indeed, it is plausible that, by neutralizing virus, the beneficial convalescent plasma caused a drop of viral load in respiratory fluids, effectively dampening IFN responses and the downstream noxious effects of IFN hyperactivation (39). These include dysregulation of complement and coagulation functions, potentially leading to lung damage and respiratory failure (45). Lung damage might be further compounded by dysfunctional ECM receptors and focal adhesion pathways, as revealed by plasma proteomics, which may underlie the lung injury seen in severe and fatal cases of COVID-19 (33). Plasma factors and immune cell types partaking in the changes observed post-transfusion likely identify cellular mechanisms critically associated with disease resolution. The characterization of the temporal dynamics of biological processes associated with COVID-19 resolution may identify targets of therapeutic interventions against severe COVID-19, even beyond convalescent plasma therapy. Furthermore, since disease resolution in the study patient was associated with convalescent plasma transfusion, we also characterized key properties of donor plasma, such as virus neutralizing capacity, which are too often ignored when assessing the value of convalescent plasma therapy for COVID-19, as previously discussed (15).

Our study has limitations. One is the study of a single patient. The particular characteristics of the patient, including the underlying immune dysfunction, undoubtedly played a role in both the clinical outcome of the convalescent plasma donation and the ways in which disease resolution occurred. However, most of our findings are corroborated by the rapidly evolving knowledge of the pathways associated with COVID-19 disease and its severity, as presented in *Results*. Thus, we are reasonably confident that our findings are applicable beyond the particular patient under study. A second limitation of our work lies in our inability – despite multiple attempts – to access the plasma obtained from the first, anonymous donor, which had no beneficial effect on the recipient's disease. A comparison between the two sets of convalescent plasma samples might have shed light on key therapeutic properties of convalescent plasma for COVID-19. Thirdly, we do not know which organ/cell(s) the viral transcripts we detected in plasma are derived from. Care of the patient required no organ biopsies, and we found no viral RNA in peripheral blood cells (not shown). Nevertheless, our data strongly point to RNAemia as a proxy for severe infection, as also proposed by others (46), and perhaps as a tool to monitor viral clearance systemically to identify cases of post-acute viral persistence, which may potentially lead to protracted symptoms (47).

## Ethics Statement

We confirm that all relevant ethical guidelines have been followed and all study activities were approved by the Rutgers Institutional Review Board (Pro2020000655). We also confirm that written consent to publish the present work was obtained from the patient. The signed consent is available for review by the editorial team. The patients/participants provided their written informed consent to participate in this study.

## Author Contributions

VG, RU, SL, MG, and NB: conceptualization. RD and ST: methodology. JY, RD, and NB: formal analysis. RU, VG, RD, JV, NB, and PM: investigation. AO, DH, MC, SG, MP, CP, AP, and SL: resources. JV, NB, VG, RU, RD, ST, JY, and MG: data curation. NB and MG: writing—original draft. MG, NB, VG, RU, RD, JY, SG, MP, SL, and CP: writing—review and editing. NB, RU, VG, RD, and JY: visualization. MG and SL: supervision. JY and MG: funding. All authors contributed to the article and approved the submitted version.

## Funding

This work was funded by NIH grants R01 HL149450, R01 HL149450-S1, R61 HD105619, R01 AI158911, U01 AI122285-S1, UL1 TR003017, R00 GM118907, and R01 CA227291. Work performed at the Rutgers Biological Mass Spectrometry Facility was partially supported by NIH S10 OD025140.

## Conflict of Interest

The authors declare that the research was conducted in the absence of any commercial or financial relationships that could be construed as a potential conflict of interest.

## Publisher's Note

All claims expressed in this article are solely those of the authors and do not necessarily represent those of their affiliated organizations, or those of the publisher, the editors and the reviewers. Any product that may be evaluated in this article, or claim that may be made by its manufacturer, is not guaranteed or endorsed by the publisher.

## References

[1] Merad M Blish CA Sallusto F Iwasaki A. The immunology and immunopathology of COVID-19. Science. (2022) 375:1122–7. 10.1126/science.abm8108 35271343 PMC12828912

[2] Carvalho T Krammer F Iwasaki A. The first 12 months of COVID-19: a timeline of immunological insights. Nat Rev Immunol. (2021) 21:245–56. 10.1038/s41577-021-00522-1 33723416 PMC7958099

[3] Zhang Q Bastard P Liu Z Le Pen J Moncada-Velez M Chen J. Inborn errors of type I IFN immunity in patients with life-threatening COVID-19. Science. (2020) 370:eabd4570. 10.1126/science.abd4570 32972995 PMC7857407

[4] Bastard P Rosen LB Zhang Q Michailidis E Hoffmann HH Zhang Y. Autoantibodies against type I IFNs in patients with life-threatening COVID-19. Science. (2020) 370:eabd4585. 10.1126/science.abd4585 32972996 PMC7857397

[5] Mathew D Giles JR Baxter AE Oldridge DA Greenplate AR Wu JE. Deep immune profiling of COVID-19 patients reveals distinct immunotypes with therapeutic implications. Science. (2020) 369:eabc8511. 10.1126/science.abc8511 32669297 PMC7402624

[6] Lucas C Wong P Klein J Castro TBR Silva J Sundaram M. Longitudinal analyses reveal immunological misfiring in severe COVID-19. Nature. (2020) 584:463–9. 10.1038/s41586-020-2588-y 32717743 PMC7477538

[7] Kaneko N Kuo HH Boucau J Farmer JR Allard-Chamard H Mahajan VS. Loss of Bcl-6-expressing T follicular helper cells and germinal centers in COVID-19. Cell. (2020) 183:143–57.e13. 10.1016/j.cell.2020.08.025 32877699 PMC7437499

[8] Schulte-Schrepping J Reusch N Paclik D Baßler K Schlickeiser S Zhang B. Severe COVID-19 is marked by a dysregulated myeloid cell compartment. Cell. (2020) 182:1419–40.e23. 10.1016/j.cell.2020.08.001 32810438 PMC7405822

[9] Silvin A Chapuis N Dunsmore G Goubet AG Dubuisson A Derosa L. Elevated calprotectin and abnormal myeloid cell subsets discriminate severe from mild COVID-19. Cell. (2020) 182:1401–18.e18. 10.1016/j.cell.2020.08.002 32810439 PMC7405878

[10] Chang SE Feng A Meng W Apostolidis SA Mack E Artandi M. New-onset IgG autoantibodies in hospitalized patients with COVID-19. Nat Commun. (2021) 12:5417. 10.1038/s41467-021-25509-3 34521836 PMC8440763

[11] Wang EY Mao T Klein J Dai Y Huck JD Jaycox JR. Diverse functional autoantibodies in patients with COVID-19. Nature. (2021) 595:283–288. 10.1038/s41586-021-03631-y 34010947 PMC13130511

[12] Chakraborty S Gonzalez J Edwards K Mallajosyula V Buzzanco AS Sherwood R. Proinflammatory IgG Fc structures in patients with severe COVID-19. Nat Immunol. (2021) 22:67–73. 10.1038/s41590-020-00828-7 33169014 PMC8130642

[13] Böhm I. Decrease of B-cells and autoantibodies after low-dose methotrexate. Biomed Pharmacother. (2003) 57:278–81. 10.1016/S0753-3322(03)00086-6 14499173

[14] Bonelli MM Mrak D Perkmann T Haslacher H Aletaha D. SARS-CoV-2 vaccination in rituximab-treated patients: evidence for impaired humoral but inducible cellular immune response. Ann Rheum Dis. (2021) 80:1355–1356. 10.1136/annrheumdis-2021-220408 33958323

[15] Honjo K Russell RM Li R Liu W Stoltz R Tabengwa EM. Convalescent plasma-mediated resolution of COVID-19 in a patient with humoral immunodeficiency. Cell Rep Med. (2021) 2:100164. 10.1016/j.xcrm.2020.100164 33521696 PMC7817775

[16] Kuleshov MV Jones MR Rouillard AD Fernandez NF Duan Q Wang Z. Enrichr: a comprehensive gene set enrichment analysis web server 2016 update. Nucleic Acids Res. (2016) 44:W90–7. 10.1093/nar/gkw377 27141961 PMC4987924

[17] Ramlall V Thangaraj PM Meydan C Foox J Butler D Kim J. Immune complement and coagulation dysfunction in adverse outcomes of SARS-CoV-2 infection. Nat Med. (2020) 26:1609–15. 10.1038/s41591-020-1021-2 32747830 PMC7809634

[18] Lo MW Kemper C Woodruff TM. COVID-19: complement, coagulation, and collateral damage. J Immunol. (2020) 205:1488–95. 10.4049/jimmunol.2000644 32699160 PMC7484432

[19] Ricklin D Reis ES Lambris JD. Complement in disease: a defence system turning offensive. Nat Rev Nephrol. (2016) 12:383–401. 10.1038/nrneph.2016.70 27211870 PMC4974115

[20] Semeraro N Colucci M. The prothrombotic state associated with SARS-CoV-2 infection: pathophysiological aspects. Mediterr J Hematol Infect Dis. (2021) 13:e2021045. 10.4084/MJHID.2021.045 34276914 PMC8265369

[21] Gómez-Rial J Currás-Tuala MJ Rivero-Calle I Gómez-Carballa A Cebey-López M Rodríguez-Tenreiro C. Increased serum levels of sCD14 and sCD163 indicate a preponderant role for monocytes in COVID-19 immunopathology. Front Immunol. (2020) 11:560381. 10.3389/fimmu.2020.560381 33072099 PMC7538662

[22] Zhu P Lv C Fang C Peng X Sheng H Xiao P. Heat shock protein member 8 is an attachment factor for infectious bronchitis virus. Front Microbiol. (2020) 11:1630. 10.3389/fmicb.2020.01630 32765462 PMC7381282

[23] Taguwa S Yeh MT Rainbolt TK Nayak A Shao H Gestwicki JE. Zika virus dependence on host Hsp70 provides a protective strategy against infection and disease. Cell Rep. (2019) 26:906–920.e3. 10.1016/j.celrep.2018.12.095 30673613 PMC6709865

[24] Ramos CHI Ayinde KS. Are Hsp90 inhibitors good candidates against Covid-19? Curr Protein Pept Sci. (2020). 10.2174/1389203721666201111160925 33176644

[25] Paladino L Vitale AM Caruso Bavisotto C Conway de Macario E Cappello F Macario AJL. The role of molecular chaperones in virus infection and implications for understanding and treating COVID-19. J Clin Med. (2020) 9:3518. 10.3390/jcm9113518 33143379 PMC7693988

[26] Murdaca G Contini P Cagnati P Marenco S Pieri G Lantieri F. Behavior of soluble HLA-A, -B, -C and HLA-G molecules in patients with chronic hepatitis C virus infection undergoing pegylated interferon-α and ribavirin treatment: potential role as markers of response to antiviral therapy. Clin Exp Med. (2017) 17:93–100. 10.1007/s10238-015-0399-5 26567007

[27] Cooper EH Forbes MA Hambling MH. Serum beta 2-microglobulin and C reactive protein concentrations in viral infections. J Clin Pathol. (1984) 37:1140–3. 10.1136/jcp.37.10.1140 6092437 PMC498955

[28] Conca W Alabdely M Albaiz F Foster MW Alamri M Alkaff M. Serum β2-microglobulin levels in Coronavirus disease 2019 (Covid-19): another prognosticator of disease severity? PLoS ONE. (2021) 16:e0247758. 10.1371/journal.pone.0247758 33647017 PMC7920360

[29] Owusu BY Zimmerman KA Murphy-Ullrich JE. The role of the endoplasmic reticulum protein calreticulin in mediating TGF-β-stimulated extracellular matrix production in fibrotic disease. J Cell Commun Signal. (2018) 12:289–99. 10.1007/s12079-017-0426-2 29080087 PMC5842189

[30] Echavarría-Consuegra L Cook GM Busnadiego I Lefèvre C Keep S Brown K. Manipulation of the unfolded protein response: a pharmacological strategy against coronavirus infection. PLoS Pathog. (2021) 17:e1009644. 10.1371/journal.ppat.1009644 34138976 PMC8211288

[31] van de Veerdonk FL Netea MG van Deuren M van der Meer JW de Mast Q Brüggemann RJ. Kallikrein-kinin blockade in patients with COVID-19 to prevent acute respiratory distress syndrome. Elife. (2020) 9:e57555. 10.7554/eLife.57555.sa2 32338605 PMC7213974

[32] Bonnans C Chou J Werb Z. Remodelling the extracellular matrix in development and disease. Nat Rev Mol Cell Biol. (2014) 15:786–801. 10.1038/nrm3904 25415508 PMC4316204

[33] Leng L Cao R Ma J Mou D Zhu Y Li W. Pathological features of COVID-19-associated lung injury: a preliminary proteomics report based on clinical samples. Signal Transduct Target Ther. (2020) 5:240. 10.1038/s41392-020-00355-9 33060566 PMC7557250

[34] Actis Dato V Chiabrando GA. The role of low-density lipoprotein receptor-related protein 1 in lipid metabolism, glucose homeostasis and inflammation. Int J Mol Sci. (2018) 19:1780. 10.3390/ijms19061780 29914093 PMC6032055

[35] Mantuano E Brifault C Lam MS Azmoon P Gilder AS Gonias SL. LDL receptor-related protein-1 regulates NFκB and microRNA-155 in macrophages to control the inflammatory response. Proc Natl Acad Sci USA. (2016) 113:1369–74. 10.1073/pnas.1515480113 26787872 PMC4747752

[36] Begue F Tanaka S Mouktadi Z Rondeau P Veeren B Diotel N. Altered high-density lipoprotein composition and functions during severe COVID-19. Sci Rep. (2021) 11:2291. 10.1038/s41598-021-81638-1 33504824 PMC7841145

[37] Stuart T Butler A Hoffman P Hafemeister C Papalexi E Mauck WM 3rd. Comprehensive integration of single-cell data. Cell. (2019) 177:1888–1902.e21. 10.1016/j.cell.2019.05.031 31178118 PMC6687398

[38] Lee AJ Ashkar AA. The dual nature of type I and type II interferons. Front Immunol. (2018) 9:2061. 10.3389/fimmu.2018.02061 30254639 PMC6141705

[39] Lee JS Shin EC. The type I interferon response in COVID-19: implications for treatment. Nat Rev Immunol. (2020) 20:585–586. 10.1038/s41577-020-00429-3 32788708 PMC8824445

[40] Cantaert T Baeten D Tak PP van Baarsen LG. Type I IFN and TNFα cross-regulation in immune-mediated inflammatory disease: basic concepts and clinical relevance. Arthritis Res Ther. (2010) 12:219. 10.1186/ar3150 21062511 PMC2991015

[41] Wilk AJ Lee MJ Wei B Parks B Pi R Martínez-Colón GJ. Multi-omic profiling reveals widespread dysregulation of innate immunity and hematopoiesis in COVID-19. J Exp Med. (2021) 218:e20210582. 10.1084/jem.20210582 34128959 PMC8210586

[42] Lee JS Park S Jeong HW Ahn JY Choi SJ Lee H. Immunophenotyping of COVID-19 and influenza highlights the role of type I interferons in development of severe COVID-19. Sci Immunol. (2020) 5:eabd1554. 10.1126/sciimmunol.abd1554 32651212 PMC7402635

[43] Hadjadj J Yatim N Barnabei L Corneau A Boussier J Smith N. Impaired type I interferon activity and inflammatory responses in severe COVID-19 patients. Science. (2020) 369:718–724. 10.1126/science.abc6027 32661059 PMC7402632

[44] Blanco-Melo D Nilsson-Payant BE Liu WC Uhl S Hoagland D Møller R. Imbalanced host response to SARS-CoV-2 drives development of COVID-19. Cell. (2020) 181:1036–45.e9. 10.1016/j.cell.2020.04.026 32416070 PMC7227586

[45] Frantzeskaki F Armaganidis A Orfanos SE. Immunothrombosis in acute respiratory distress syndrome: cross talks between inflammation and coagulation. Respiration. (2017) 93:212–25. 10.1159/000453002 27997925

[46] Gutmann C Takov K Burnap SA Singh B Ali H Theofilatos K. SARS-CoV-2 RNAemia and proteomic trajectories inform prognostication in COVID-19 patients admitted to intensive care. Nat Commun. (2021) 12:3406. 10.1038/s41467-021-23494-1 34099652 PMC8184784

[47] Huang Z Ning B Yang HS Youngquist BM Niu A Lyon CJ. Sensitive tracking of circulating viral RNA through all stages of SARS-CoV-2 infection. J Clin Invest. (2021) 131:e146031. 10.1172/JCI146031 33561010 PMC8011898

