# Supplementary material for: Longitudinal Analysis of Biologic Correlates of COVID-19 Resolution: Case Report
Source: Front Med (Lausanne). 2022 Jun 15;9:915367. doi: 10.3389/fmed.2022.915367 (PMC9240225; doi:10.3389/fmed.2022.915367)
Supplement: Supplementary file 1 [file Data_Sheet_1.DOCX]

**Supplemental Figures**

**
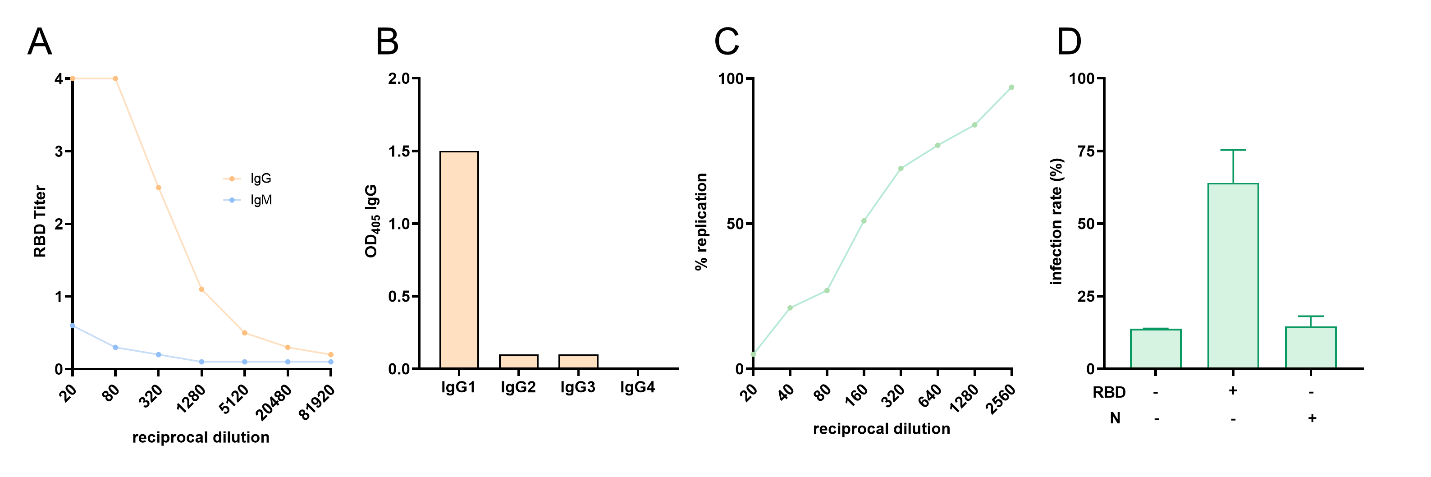
Supplemental Figure 1. Antibody profiles of the second convalescent plasma donor (patient’s relative). (A)** ELISA endpoint titers for SARS-CoV-2 RBD-specific IgG and IgM. **(B)** Levels of IgG subclasses targeting RBD. **(C)** Neutralizing activity of donor plasma was determined utilizing ACE2-expressing HeLa cells and mNeonGreen-tagged SARS-CoV-2 virus. The neutralizing titer was expressed as NT50 (reciprocal dilution of plasma yielding 50% neutralization). **(D)** Donor plasma was depleted of either RBD- or N-specific antibodies by pre-incubating plasma aliquots with either recombinant RBD or N (Nucleocapsid) proteins and then testing them for neutralizing activity.


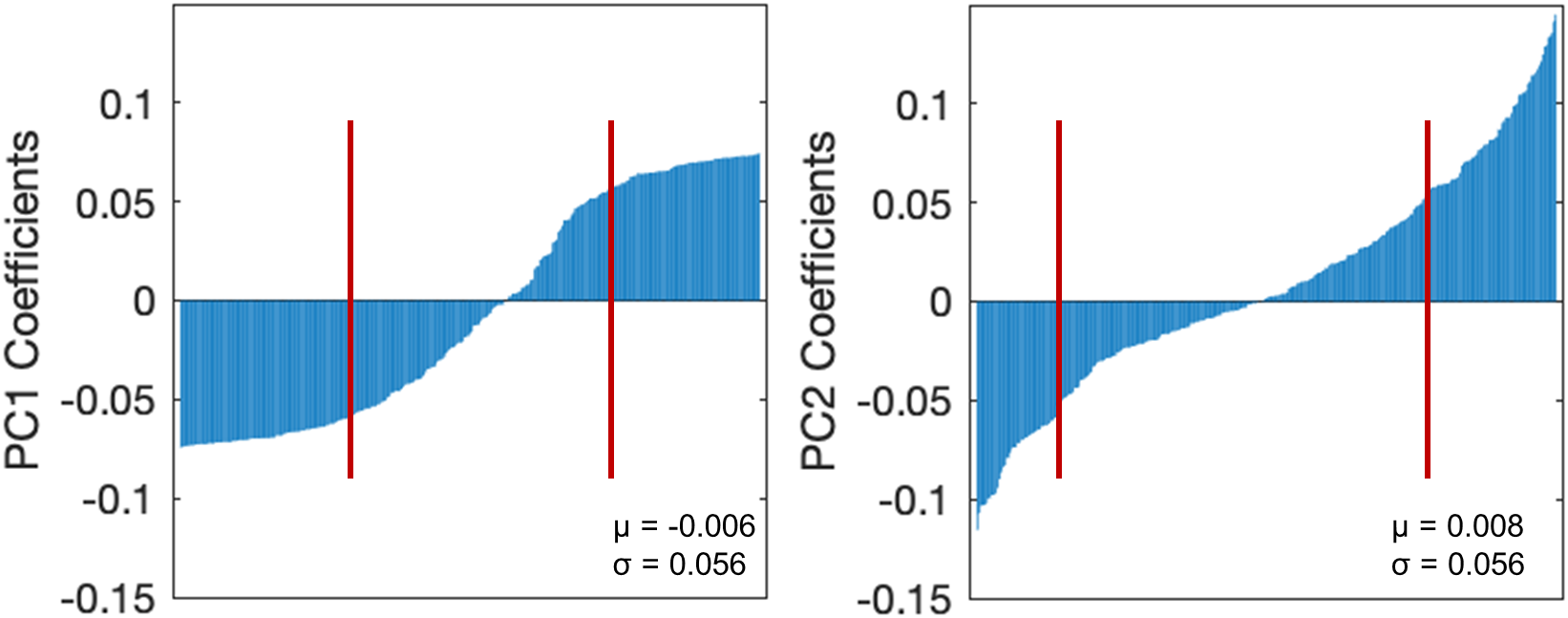


**B**

A

**Supplemental Figure 2.** Coefficients for proteins from Principal Component 1 (PC1) from the proteomic analyses (Figure 2A). Proteins with decreasing expression after convalescent plasma transfusion (Module A, orange arrow) possessed coefficients greater than one standard deviation from the mean, while proteins with increasing expression (Module B, green arrow) possessed coefficients less than one standard deviation from the mean.
