# Supplementary material for: Longitudinal Analysis of Biologic Correlates of COVID-19 Resolution: Case Report
Source: Front Med (Lausanne). 2022 Jun 15;9:915367. doi: 10.3389/fmed.2022.915367 (PMC9240225; doi:10.3389/fmed.2022.915367)
Supplement: Supplementary file 2 [file Data_Sheet_2.docx]

**Materials and Methods**

**Subject Recruitment**

All study activities were approved by the Rutgers Institutional Review Board (Pro2020000655). Informed consent was obtained from the patient in her 50’s (51-55 age range, Caucasian, female) and a relative in their 46-50 age range (Caucasian, male). Blood samples were collected from the two participants at times indicated in the text and in Figure 1A.

**Antibody binding by enzyme-linked immunosorbent assay (ELISA)**

Antibody binding was performed by ELISA utilizing SARS-CoV-2 receptor binding domain (RBD) of the Spike protein as solid-phase antigen, as described (1). HRP-conjugated mouse anti-human IgG1, IgG2, IgG3, and IgG4 (Southern Biotech, Birmingham, AL, USA) secondary antibodies were used at 1:2,000 dilution. Each sample was tested in duplicate. End-point titers were calculated using background-subtracted data and an established cut-off (1).

**Absorption of convalescent plasma with SARS-CoV-2 antigens**

96-well ELISA plates (Nunc MaxiSorp, Thermofisher, Rochester, NY, USA) were coated with 500 ng/well of SARS-CoV-2 RBD or N protein at 4°C overnight. Coated plates were washed three times with washing buffer (PBS containing 0.05% Tween 20) (Sigma-Aldrich, St. Louis, MO, USA) blocked with PBS containing 1% BSA (Sigma-Aldrich, St. Louis, MO, USA) for 30 min at 37°C. After washing, plasma samples were diluted 1:10 in PBS containing 1% BSA (Sigma-Aldrich, St. Louis, MO, USA) and incubated overnight at 4°C. Absorption was repeated at least four times utilizing fresh antigen-coated plates at each cycle. To monitor depletion of antigen-specific antibodies prior to use in neutralization assays, antigen-specific IgG titers of untreated and absorbed samples were determined as described above.

**Cell lines**

Vero E6 were obtained from the American Type Culture Collection (ATCC) (Manassas, VA, USA); HeLa cells stably expressing ACE2 (HeLa-ACE2) were obtained from Dennis Burton at the Scripps Research Institute (2). All cell lines were maintained in high-glucose Dulbecco’s modified Eagle’s medium (DMEM; Corning, Corning, NY, USA) supplemented with 10% fetal bovine serum (FBS; Seradigm, Radnor, PA, USA), 2mM L-glutamine, and 1% penicillin/streptomycin (Corning, Corning, NY, USA), and incubated in humidified atmospheric air containing 5% CO2 at 37°C.

**SARS-CoV-2 virus**

The virus stock of mNeonGreen (mNG) SARS-CoV-2 was obtained from Pei-Yong Shi at the University of Texas Medical Branch at Galveston. The virus stock was produced using the virus isolate of the first patient diagnosed in the USA, in which the ORF7 of the viral genome was replaced with the reporter mNG gene (3). Propagation of viral stocks was performed with Vero E6 cells using DMEM supplemented with 2% FBS. The virus titers were determined by standard plaque assay utilizing Vero E6 cells and recorded as plaque forming units per milliliter (PFU/mL).

**SARS-CoV-2 neutralization assay**

HeLa-Ace2 cells were seeded in 96-well black optical-bottom plates at a density of 1 × 10^4^ cells/well in FluoroBrite DMEM (Thermo Fisher Scientific, Waltham, MA, USA) containing 4% FBS (Seradigm, Radnor, PA, USA), 2mM L-glutamine, and 1% penicillin/streptomycin (Corning, NY, USA), and incubated overnight at 37°C with 5% CO2. On the following day, each sample was subjected to two-fold serial dilution in DMEM without FBS, and incubated with mNG SARS-CoV-2 at 37°C for 1.5 hrs. The virus-plasma mixture was transferred to 96-well plates containing Hela-Ace2 cells at a final multiplicity of infection (MOI) of 0.25 (viral PFU:cell). For each sample, the starting dilution was 1:20 and the final dilution of 1:10,240. After incubating infected cells at 37°C for 20 hrs, mNG SARS-CoV-2 fluorescence was measured using a Cytation^TM^ 5 reader (BioTek, Winooski, VT, USA). Each sample was tested in duplicate. Relative fluorescent units were converted to percent neutralization by normalizing the sample-treatment to non-sample-treatment controls and plotting the data with a nonlinear regression curve fit to determine the titer neutralizing 50% of SARS-CoV-2 fluorescence (NT50).

**Buffy coat isolation and storage**

All buffy coats were extracted on the day of receipt at the research laboratory. Briefly, blood tube was centrifuged at 800 × g for 15 mins and plasma was carefully removed, aliquoted, and stored at −80°C. Buffy coat layer was collected, and red blood cells (RBCs) were lysed by incubation in 1× BD Pharm Lyse lysing solution (BD Biosciences, San Jose, CA, USA) at room temperature for 10 mins. Cells were washed 3 times by centrifugation with PBS and counted using a hemocytometer. Buffy coats were cryopreserved in liquid nitrogen in FBS containing 10% dimethyl sulfoxide (DMSO, Thermo Fisher Scientific, Waltham, MA, USA) and stored until use.

**Plasma RT-PCR**

Briefly, genomic RNA was extracted from plasma using the RNeasy plus mini kit (QIAGEN, Hilden, Germany). SARS-CoV-2 RNA for nucleocapsid (N) and RNA-dependent RNA polymerase (RdRp) genes was detected by RT-PCR assay, and the results were presented as cycle threshold (Ct).

**Cytokines**

Cytokine analysis was performed by the Immune Monitoring and Advanced Genomics Core Facility at Rutgers University using a Luminex® Discovery Assay (R&D System Inc., Minneapolis, MN, USA). The Human Premixed Multi-Analyte Kits were used to detect 48 cytokine/chemokines (CD40L, EGF, Eotaxin, FGF-2, LT-3L, Fractalkine, G-CSF, GROa, IFNα2, IFNɣ, IL-1α, IL-1β, IL-1RA, IL-2, IL3, IL-4, IL-5, IL-6, IL-7, IL8, IL-9, IL-10, IL-12 (p40), IL-12 (p70), IL-13, IL-15, IL17A, IL-17E, IL-17F, IL-18, IL-22, IL-27, IP-10, MCP-1, MCP3, M-CSF, MDC, MIG, MIP-1α, MIP-1β, PDGF-AA, PDGF-AB, RANTES, TGFα, TNFα, TNFβ, VEGF-A) according to the manufacturer’s recommendations. Assays were performed in microtiter plates and plates were read with the Luminex 200 instrument. Each sample was measured in duplicate.

**Proteomics**

Proteomic analysis was performed by the Rutgers Biological Mass Spectrometry Facility. Plasma samples were prepared for mass spectrometry proteome analysis before or after immunodepletion of abundant plasma proteins. Abundant proteins were removed from 10µl samples using an Agilent Human 14 Multiple Affinity Removal Spin (MARS) Cartridge (Agilent, Santa Clara, CA, USA) using manufacturer’s methods. Protein microchemistry, mass spectrometry and data processing were conducted as described previously (4). In brief, protein concentrations of each sample were determined and equal amounts of each (before and after immunodepletion) were proteolytically digested using both filter aided sample preparation (5) (FASP) or in-gel protocols as described previously (4, 6); thus four independent analyses were conducted. Peptides were labeled with TMT 11plex isobaric reagents (Thermo Fisher Scientific, Waltham, MA, USA), pooled then prefractionated by alkaline reverse phase HPLC (4). Reporter ions in individual fractions were measured using synchronous precursor selection MS3 methods on a Thermo Eclipse Tribrid mass spectrometer (Thermo Fisher Scientific, Waltham, MA, USA). Peak lists were generated using Proteome Discoverer 2.2 and data were searched using a local implementation of the Global Proteome Machine (7). Reporter ion intensities were extracted using in-house scripts (https://github.com/cgermain/IDEAA). Data were normalized to the total reporter ion intensity per channel for each of the four independent analyses to account for differences in protein amounts or labeling efficiency for each sample. Reporter ion intensities were measured after spectra were filtered to remove spectra corresponding to peptides that are non-tryptic or not fully digested, peptides that are incompletely labeled or labeled at positions other than n-termini and lysine, or peptides that contain post-translational modifications that introduce variability

**Multivariate analysis**

Plasma cytokine and protein expression measurements were first pre-processed and standardized in MATLAB 2020b (Mathworks; Natick, MA) before subsequent hierarchical clustering and principal component analyses. From the initial set of 48 cytokines and chemokines in the Human Premixed Multi-Analyte Kit, 18 cytokines and chemokines were removed due to poor data quality, where the analyte either fell below the limit of detection or were otherwise unmeasurable in at least one of the samples (EGF, IFNα2, IFNɣ, IL-2, IL-3, IL-4, IL-7, IL-10, IL-13, M-CSF, RANTES, TNFβ). Remaining cytokine measurements were log_10_-transformed and standardized by Z-score. Hierarchical clustering analysis and principal component analysis were performed in MATLAB on the standardized cytokine and protein expression measurements using custom scripts.

**Single cell RNA sequencing**

Cells were washed in complete RPMI medium (supplemented with 10% FBS, 2mM L-glutamine, and 1% penicillin/streptomycin), counted, and assessed for viability using a Countess II automated cell counter (Invitrogen, [Waltham, MA](https://nam02.safelinks.protection.outlook.com/?url=https%3A%2F%2Fwww.google.com%2Fsearch%3Frlz%3D1C1CHBF_enUS921US921%26q%3DWaltham%26stick%3DH4sIAAAAAAAAAOPgE-LUz9U3MDNLKUxS4gAxM6qMTbW0spOt9POL0hPzMqsSSzLz81A4VhmpiSmFpYlFJalFxYtY2cMTc0oyEnN3sDICANGzN1FQAAAA%26sa%3DX%26ved%3D2ahUKEwj8woO785r0AhXuc98KHRgUBLMQmxMoAXoECE4QAw&data=04%7C01%7Cvg238%40njms.rutgers.edu%7Cb281b30ce7014548b07808d9a9ec167b%7Cb92d2b234d35447093ff69aca6632ffe%7C1%7C0%7C637727653711146044%7CUnknown%7CTWFpbGZsb3d8eyJWIjoiMC4wLjAwMDAiLCJQIjoiV2luMzIiLCJBTiI6Ik1haWwiLCJXVCI6Mn0%3D%7C3000&sdata=I3JrwMp3q6oMWfBqx79vBOgTMtz5CcN6bRTt9nRWlDs%3D&reserved=0), USA). Cells were then suspended to 1 × 10^6^/ml in complete RPMI for single-cell emulsion preparation. scRNA-seq GEX libraries were prepared by the Immune Monitoring and Advanced Genomics Rutgers Core Facility according to 10X Genomics specifications. Independent cell suspensions were loaded for droplet-encapsulation by the Chromium Controller (10X Genomics). Single-cell cDNA synthesis, amplification, and sequencing libraries were generated using the Single Cell 5′ Reagent kit (10X Genomics, Pleasanton, CA, USA) following the manufacturer’s instructions. The libraries were shipped to Novogene and sequenced with the Illumina NovaSeq 6000 platform (200 M, 150 bp paired-end reads).

Raw sequencing data were processed using the CellRanger software (version 3.1.0). Reads were aligned to a custom reference genome created with the reference human genome (GRCh38) and SARS-CoV-2 reference genome (NC_045512.2). The resulting unique molecular identifier (UMI) count matrices were imported into R (version 4.0.2) and processed with the R package Seurat (version 4.0.0) (8). The data was normalized using SCTransform (9) and then dimensionality reduced by finding the top 50 principal components and then using these principal components to run UMAP (10). Cell types were identified using Seurat’s MapQuery function, along with the PBMC scRNA-seq datasets (8). To account for overclustering, most likely due to variable TCR gene expression in the T cell populations, all TCR genes were removed, the analysis and mapping were repeated, and more homogenous cell populations obtained after mapping. Differential expression results were similar with and without the TCR genes. Doublet analyses were performed using R package Scrublet (11). MAST package (version 1.8.2) (12) were to identify differential expressing genes across different cell populations. Differentially expressing genes were used with the fast pre-ranked gene set enrichment analysis (fGSEA) package in R for the hallmark pathways (13).

**References**

1. Datta P, Ukey R, Bruiners N, Honnen W, Carayannopoulos MO, Reichman C, et al. Highly versatile antibody binding assay for the detection of SARS-CoV-2 infection and vaccination. J Immunol Methods. 2021;499:113165.

2. Rogers TF, Zhao F, Huang D, Beutler N, Burns A, He WT, et al. Isolation of potent SARS-CoV-2 neutralizing antibodies and protection from disease in a small animal model. Science. 2020;369(6506):956-63.

3. Xie X, Muruato A, Lokugamage KG, Narayanan K, Zhang X, Zou J, et al. An Infectious cDNA Clone of SARS-CoV-2. Cell Host Microbe. 2020;27(5):841-8.e3.

4. Tannous A, Boonen M, Zheng H, Zhao C, Germain CJ, Moore DF, et al. Comparative Analysis of Quantitative Mass Spectrometric Methods for Subcellular Proteomics. J Proteome Res. 2020;19(4):1718-30.

5. Wiśniewski JR. Quantitative Evaluation of Filter Aided Sample Preparation (FASP) and Multienzyme Digestion FASP Protocols. Anal Chem. 2016;88(10):5438-43.

6. Sleat DE, Della Valle MC, Zheng H, Moore DF, Lobel P. The mannose 6-phosphate glycoprotein proteome. J Proteome Res. 2008;7(7):3010-21.

7. Beavis RC. Using the global proteome machine for protein identification. Methods Mol Biol. 2006;328:217-28.

8. Hao Y, Hao S, Andersen-Nissen E, Mauck WM, 3rd, Zheng S, Butler A, et al. Integrated analysis of multimodal single-cell data. Cell. 2021;184(13):3573-87.e29.

9. Hafemeister C, Satija R. Normalization and variance stabilization of single-cell RNA-seq data using regularized negative binomial regression. Genome Biol. 2019;20(1):296.

10. Becht E, McInnes L, Healy J, Dutertre CA, Kwok IWH, Ng LG, et al. Dimensionality reduction for visualizing single-cell data using UMAP. Nat Biotechnol. 2018:doi: 10.1038/nbt.4314.

11. Wolock SL, Lopez R, Klein AM. Scrublet: Computational Identification of Cell Doublets in Single-Cell Transcriptomic Data. Cell Syst. 2019;8(4):281-91.e9.

12. Finak G, McDavid A, Yajima M, Deng J, Gersuk V, Shalek AK, et al. MAST: a flexible statistical framework for assessing transcriptional changes and characterizing heterogeneity in single-cell RNA sequencing data. Genome Biol. 2015;16:278.

13. Liberzon A, Birger C, Thorvaldsdóttir H, Ghandi M, Mesirov JP, Tamayo P. The Molecular Signatures Database (MSigDB) hallmark gene set collection. Cell Syst. 2015;1(6):417-25.
